# Understanding the Competitive Advantages of Car Sharing from the Travel-Cost Perspective

**DOI:** 10.3390/ijerph17134666

**Published:** 2020-06-29

**Authors:** Xianlei Dong, Yongfang Cai, Jiaming Cheng, Beibei Hu, Huijun Sun

**Affiliations:** 1 School of Business, Shandong Normal University, Jinan 250358, China; dongxianlei@sdnu.edu.cn (X.D.); 201724040209@stu.sdnu.edu.cn (Y.C.); 201724040218@stu.sdnu.edu.cn (J.C.); 2 Key Laboratory of Transport Industry of Big Data Application Technologies for Comprehensive Transport, Ministry of Transport, Beijing Jiaotong University, Beijing 100044, China; hjsun1@bjtu.edu.cn

**Keywords:** car sharing, taxi, GPS trajectory data, travel-cost advantage comparison

## Abstract

The emergence and development of car sharing can not only satisfy people’s diverse travel demands, but also can bring a new solution to facilitate urban low-carbon and green development. With the increasing acceptance of car sharing, the market competition between car sharing and traditional taxis is becoming increasingly fierce. Therefore, we explore the advantages of car sharing to travelers compared with taxis. In this paper, we first use the GPS (Global Positioning System) trajectory data of car sharing orders to construct a comparative advantage model based on travel-cost. Then, we take Beijing as the research area to explore the travel-cost advantages of car sharing in terms of the time and space dimensions compared with taxis, through calculating the travel-cost of car sharing and using simulation to calculate that of taxis. The results of the comparison between car sharing and taxis from the perspective of travel-cost are as follows: (1) Compared with short trips, the travel-cost advantage of car sharing is relatively higher in medium and long trips; for travelers, the taxi has a higher travel-cost advantage when the travel time is either very long or very short. (2) On weekdays, it is more cost-effective to travel by shared cars for travelers before the rush hours in the evening, and the travel-cost advantage of using taxis is greater after the evening peak. (3) Compared with weekdays, it is more cost-effective to travel by shared cars on weekends wherever travelers are living in the main urban areas or in the remote suburbs. It is suggested that relevant departments should understand the travelers’ preference and analyze the influence mechanism of other various factors on the market demand for car sharing as per the focus on the market on the travel-cost advantages of car sharing, so as to promote the healthy and sustainable development of urban shared transportation.

## 1. Introduction

China, currently in a stage of rapid development of urbanization, has expanded the urban geographical space [1], which has resulted in distinct job–housing separation, and increased not only people’s travel time and travel distance, but also the demand for motorized travel [2]. However, due to the shortage of urban road resources, the growth space of traditional travel modes (buses and taxis) is limited, making it difficult for the traditional travel modes to meet people’s ever-increasing travel demands. Therefore, there are more and more private cars on the road. Although private cars can satisfy the travel demands of people, they exacerbate a series of environmental problems such as urban traffic jams, air pollution, energy consumption, and carbon emissions [3,4,5]. Therefore, local governments have imposed limits on the consumption and use of private cars. People’s motorized travel is still in a state where supply falls short of demand. In this situation, the emergence and development of car sharing has brought a new solution to both alleviate the traffic demand-supply imbalance in cities and promote urban sustainable development [6]. With the economic development, on one hand, people’s travel demands have increased sharply and tend to be diversified. Compared with taxis, shared cars with diverse vehicle types [7] can provide travelers with more personalized and high-quality travel options, such as business travel and personal trips for people who do not have their own cars. On the other hand, car sharing is a product of the transformation from resource-driven economy to service-driven economy. Its essence is to improve the utilization rates of automobile resources to ease the discrepancy between car ownership and traffic load capacity [8], helping facilitate urban sustainable development [9].

Currently, due to high cost of car purchase and maintenance, driving zone and license plate restriction policies, and parking spaces saturation [10], shared cars not only facilitate people’s travel, but also are gradually regarded as a viable solution to the current urban traffic problems [11]. A study has proven that car sharing can effectively address the first- and last-mile connections to public transit systems, which makes up for the shortage of current fixed public transit routes. And of all the different car sharing modes, the one-way car sharing systems show the greatest flexibility and potential in that respect [12]. Therefore, travelling by shared cars not only saves travel-cost but also is more convenient and flexible for travelers [13,14]. Moreover, as a new mode of transport, most of the shared cars are new-energy vehicles [15], which helps this mode of transport contribute significantly to reducing energy consumption and realizing low-carbon travel [16,17]. Some studies have noted that the emergence of car sharing can lower the level of car ownership and reduce car usage, thus decreasing transport-related energy consumption and greenhouse gas emissions [18,19].

Owing to these features and benefits, car sharing has quickly attracted great attention from authorities and enterprises and has begun to spread in many countries [20]. Statistically, as of October 2018, car sharing businesses operated in 47 countries on six continents, with some 32 million users sharing over 198,000 vehicles [21]. In China, car sharing conforms to the principles of innovative, coordinated, green, open and shared development, and has received strong support from national and local government departments. In order to mitigate the adverse impacts of increased auto use on urban air quality and energy security, in June 2017, the Chinese government departments solicited public opinions on “Guiding Opinions on Promoting the Healthy Development of Car Rental” and encouraged people to use information technology such as mobile internet technology and global positioning to develop car sharing models for the first time. In August 2017, “Guiding Opinions on Promoting the Healthy Development of Small Micro-coach Rental” was formally promulgated, which clearly supported the development of car sharing and pointed out that car sharing should be properly positioned so that it can coordinate with transportation modes such as buses and taxis. A lot of cities such as Beijing and Shanghai have responded to the policy. Beijing has set up car sharing stations, parking lots and charging piles in some central areas. Shanghai also builds a number of stations and charging piles for car sharing throughout the city and gives priority to ensuring people’s demand for car sharing.

The public transportation model of car sharing in China has been booming significantly due to peoples’ high demand for motorized travel and the supports from governments, but at the same time, there are already extremely popular public transportation modes (such as taxi, bus and subway) which compete with car sharing for market resources. Table 1 shows the comparison of different features among car sharing, taxi, bus and subway. As can be seen from Table 1, out of these modes, bus and subway are less comparable to car sharing because it is difficult to quantify people’s requirements on comfort, flexibility, privacy and accessibility. It would also be hard to compare the travel-costs between car sharing and other public transportation modes (bus or subway here) directly, because the travel-cost of bus and subway is much lower than that of car sharing in most cases due to the government’s transfer payment on public transportation. However, taxis, an important part of the urban passenger transport system [22,23], share a high similarity with car sharing in satisfying the motorized travel demands of people due to its similar features such as speed, comfort and flexibility. In other words, there is an obvious and great market competition between the two transport modes. Therefore, it is of great significance to study the comparative advantages of car sharing compared with taxis for grasping the reasonable market positioning of car sharing and helping car sharing and taxis develop in a coordinated manner.

This paper aims at answering the following question: What are the competitive advantages of using car sharing in terms of travel-cost for passengers, compared to taking taxis for travel? To address this issue, we build a comparative advantage model based on travel-cost according to the pricing mechanism of car sharing and taxis. Then, taking Beijing as an example, passengers’ travel-costs of using car sharing and taking taxis to travel are measured respectively, and the competitive advantages of car sharing in different scenarios are analyzed. The objective of this research is to identify the focus market of car sharing from the perspective of travel-cost, which can provide suggestion for the formulation and adjustment of car sharing policies and operational strategies, improve the rational planning of car sharing resources in cities and promote the long-term development of car sharing industry.

This study is organized as follows: Section 2 reviews previous literature. Section 3 and Section 4 describe the data and methodology, respectively. Section 5 represents the travel characteristics of car sharing users and the travel-cost advantages of car sharing in terms of the spatiotemporal dimensions. In Section 6 we compare our work with previous literature, discuss the relationships between car sharing and other transport modes and list the similarities and differences between car sharing and taxis. In Section 7 we draw our conclusions with the limitation and future works.

## 2. Literature Review

### 2.1. The Travel Behavior Characteristics of Car Sharing Users

People’s travel activities are generally limited to certain time and spaces, so their travel behavior usually presents a defined spatiotemporal distribution pattern. The characteristics of people’s travel behavior include travel mode choice, travel destination, travel distance, travel time, travel-cost and so on [24]. The study of people’s travel behavior characteristics not only is helpful to improve people’s satisfaction with travel services, but also can provide support for urban transport planning and reasonable allocation of transport resources.

Previous research on the travel behavior characteristics of residents mainly statistically analyzed people’s travel mode choice, travel distance and travel frequency using the methods of questionnaire surveys and interviews and so on [24]. For example, Dias et al. have found that the well-educated residents and those with higher incomes in the areas with great population density tend to use car sharing mode to travel [25]. Based on a mixed survey data, Efthymiou et al. constructed a hybrid choice model and confirmed that environmentally conscious people often show a much stronger behavioral intentions to car sharing [26]. By comparing the travel behavior characteristics of people with different occupations before and after the emergence of car sharing by means of questionnaire surveys and statistical analysis, Gou et al. found that more than 50% of individuals are willing to choose car sharing to travel, and this proportion is gradually increasing [27]. They also found that the travel distance of car sharing users is mostly more than 5 km each time [27].

With the advancement of mobile internet technologies and location-aware technologies, it has become easier to obtain trajectory data such as spatial trajectories of people and vehicles [28]. For such data, what people care about are the moving objects, spatial regions, temporal characteristics and moving patterns [28,29,30,31]. Studies on those aspects usually involve many trajectory parameters, including original parameters such as geographic coordinates and timestamp and derived parameters such as distance and spatial distribution [29]. In recent years, in order to discover the rich semantic information contained in trajectory data and to speculate people’s travel purposes, scholars have begun to consider the relationship between travel behavior characteristics and points of interest, and they have made good progress [28]. Qian et al. inferred that car sharing users utilize the transportation mode mainly because of commuting or business purposes, and most of the orders of car sharing are of short—to medium—distances and temporary [30]. By analyzing the frequencies of people using car sharing, Hui et al. noted that, among all the car sharing users, the regular users often choose car sharing for commuting; while the non-regular users tend to use the transportation mode for recreation and entertainment [31].

### 2.2. The Competition and Evolution of the Car Sharing Market

Car sharing, as a beneficial supplement to urban public transportation, can not only alleviate urban environmental and traffic problems such as air pollution, traffic congestion and shortage of land resources, but also meet people’s diverse travel demands. However, as a brand-new mode of car rental business, car sharing already has the key characteristics of disruptive innovation such as target market damage, low-price invasion and advantage of disruptive technology, which would cause a huge impact on traditional taxi industry [32]. Therefore, scholars began to explore the competitive advantages of car sharing from different aspects and analyze the competitive evolution trend of car sharing market [33,34,35,36,37,38,39]. Competitive advantage refers to the sustainable advantage compared with competitors, which includes cost leadership advantage and differentiation advantage [40,41].

**Figure 1:**
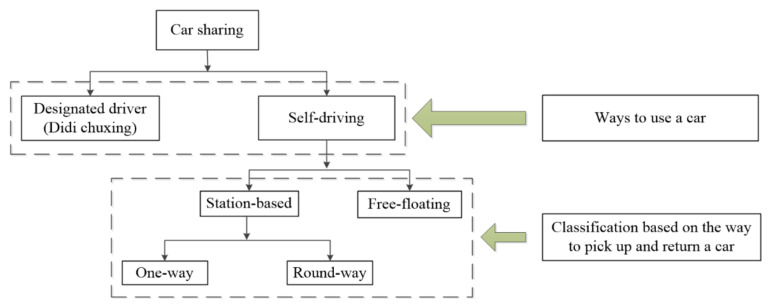
The modes of car sharing.

Currently, comparisons have been made between different modes of car sharing. Figure 1 shows existing car sharing modes with different service patterns [33]. It was found that in contrast to station-based car sharing, free-floating car sharing does not rely on the high-quality public transport infrastructure [34]. Scholars also found that compared with round-way car sharing, one-way car sharing and free-floating car sharing are more often used for short trips [35]. Compared with one-way car sharing and free-floating car sharing, the usage of round-way car sharing is much lower [35].

In addition, the game relationship between car sharing and existing transportation systems is a popular topic discussed by scholars [36,37,38,39]. Some scholars have used game theory models to explore people’s behavioral choices between the transportation modes of car sharing and private cars in different scenarios of government intervention, and they have found that people tend to choose car sharing to travel rather than private cars when the government increases the financial subsidy to car sharing [36]. Some other scholars have explored the potential of car sharing in reducing private car ownership and frequency of use of private cars, they find that 40% of car drivers would like to replace some of their private car trips by car sharing and 20% of car drivers might forego their plans to purchase a vehicle if car sharing were available [37]. As is well-known, car sharing is essentially a kind of car rental mode, but as a product of mobile internet technology and the sharing economy, car sharing is different from traditional car rental modes in many ways. For example, compared to traditional car rental modes, car sharing is more in line with people’s travel habits, thus it is more popular than the traditional car rental mode, especially when it comes to long trips within the city [38]. Besides, it is an eternal theme of sustainable transportation to generate an efficient and convenient solution for public transportation. Therefore, it is indispensable to provide co-existing opportunities for different modes of individual transportation (i.e., taxis and car sharing) [39].

To sum up, it can be found that a lot of theoretical researches and empirical analyses have been conducted. However, we notice that the current research on the competitive relationship between car sharing and traditional transport modes such as taxis is not complete. There is little research discussing the competitive advantages of car sharing compared with other traditional travel modes from the perspective of passengers’ travel-cost. Therefore, our work could enrich the literature on the field of competitive evolution of the car sharing market.

## 3. Data

### 3.1. Car Sharing in China

The global economic crisis that happened in 2007 promoted the reuse of idle resources and the rise of sharing economy. Afterwards, with the establishment and development of Airbnb and Uber, the sharing economy is flourishing around the world. In 2010, the concept of sharing economy began to sprout in China. Car sharing, a concrete application of sharing economy in the field of transportation, was introduced into China in 2011. Driven by the factors such as the slow growth of public transportation, the strong potential driving demand of residents, the increasing mature mobile internet technology and the support from governments, car sharing has been deployed in some provinces and cities of China [42].

Among those provinces and cities, car sharing was first deployed in Beijing, Shanghai and Guangzhou [42], which alleviated the heavy travel pressure and large travel demand of residents in those major cities, but meanwhile, the car sharing industry faces many urgent issues that need to be solved, as it is still in the early stages of development in China.

Beijing is the capital of China and it is the second city in terms of the number of car sharing companies [42]. Therefore, Beijing is an important study object. With the development of economics and the expansion of the city size, the job-housing separation in Beijing has become increasingly significant [43]. Statistically, around 6% of the residents live in central areas but work in suburbs; whereas 18% of the residents live in the suburbs but work in central areas [43], which increases the commuting distance of the residents. The average commuting distance in Beijing is 11.1 km [43], the longest travel distance can be more than 40 km [44]. Besides, commuting trips whose travel distance is within 5 km only account for 38%, which can be done by walking or cycling. Thus, the residents in Beijing have great commuting pressure and a certain demand for motorized travel. Statistically, the transport modal share of cars is around 24%, which is only second to walking [44], indicating that residents have high demand for travelling by car. However, due to parking and congestion problems, it has become difficult and expensive to own a car. Therefore, people have begun to rent a car instead of owning one [45]. This trend promotes the popularity of car sharing which caters to residents’ daily commuting needs to a certain extent.

Currently, the mode of car sharing in Beijing is mainly station-based. Most of the stations and charging piles are distributed within the fifth ring road. People can use mobile apps to search, reserve, open, drive and return the shared cars, and they can decide when and how long they want to use the car. However, due to the limitations such as the small number of shared cars and not being allowed to park shared cars at will, car sharing has a relatively low coverage in Beijing and is mostly used by a small number of people who live in areas within the fifth ring road. According to statistics, as of 2018, about 14% of residents in Beijing have used car sharing. Among them, only 450,000 to 500,000 people would like to use this sharing transportation mode repeatedly, accounting for 2% of the total population of Beijing. The average daily usage frequency of each shared car is 5.1 times, and the average single trip distance is 20 km [46].

### 3.2. Data Description

**Figure 2:**
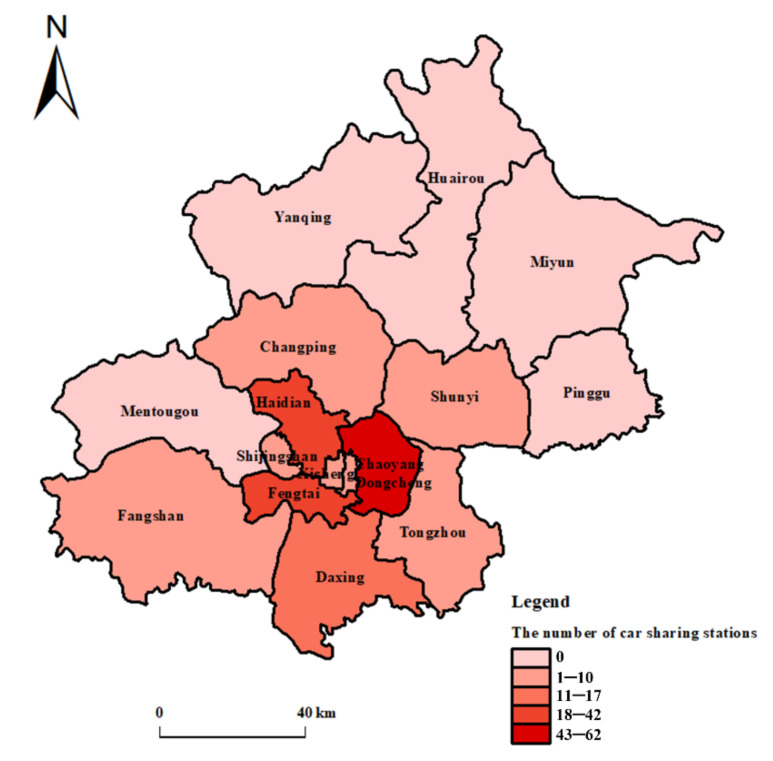
The distribution of car sharing stations in Beijing.

The data used in this paper is provided by Beijing Automotive Group, the operator of the MoreFun car sharing platform. The research data of station-based car sharing in Beijing are from 1 July 2017 to 30 September 2017. The data includes three parts:

(1) 51,250 car sharing orders. For each car sharing order, we record its order ID, pick-up time, drop-off time, pick-up station and drop-off station as shown in Table 2.
(2) The GPS (Global Positioning System) trajectory data of those car sharing orders. Each trajectory matches with a car sharing order and is represented by a series of chronologically ordered tracking records. Each of the tracking record has five data fields, including the order ID of its matching order, the time stamp corresponding to the tracking location, the coordinate longitude, the coordinate latitude and the instantaneous speed, obtained every 2 s, as shown in Table 2. A total of 76,815,720 tracking records are used in our research.
(3) The information about car sharing stations. There are 211 car sharing stations operated by MoreFun car sharing platform in Beijing. The information about these car sharing stations includes their name, coordinate longitude and latitude, as shown in Table 2. Figure 2 shows the distribution of these 211 car sharing stations.

### 3.3. Data Preprocessing

The data processing steps are as follows:

#### 3.3.1. Step 1: Data Cleaning

Data cleaning aims at eliminating abnormal and unreasonable data. Abnormal data are the car sharing orders whose drop-off time is earlier than pick-up time, or whose pick-up or drop-off stations are not included in the 211 car sharing stations mentioned in Section 3.2 due to either system errors or signal errors.

Unreasonable data refer to the car sharing orders whose travel distance is less than one kilometer or whose geographic coordinates of the GPS (Global Positioning System) tracking records are out of our research area. The geographical positioning of Beijing covers 39°26′ N to 41°03′ N and 115°25′ E to 117°30′ E. Specifically, for each car sharing order, if any of its tracking records is out of our research area, it would be deleted.

Consequently, a total of 40,802 valid car sharing orders which account for 80% of the total are kept in the dataset for further analyses after data cleaning.

#### 3.3.2. Step 2: Calculating the Travel Time and Distance for Each Car Sharing Order

First, let $P_{m}{=(P}_{m}^{1}{,P}_{m}^{2}{,\ldots ,P}_{m}^{k})$ represent the GPS tracking records for each car sharing order from the pick-up station to the drop-off station, where *m* denotes an order, and *k* denotes the number of GPS tracking records of the car sharing order *m*. Meanwhile, let $P_{m}^{n}{=(I}_{m1}^{n}{,T}_{m2}^{n}{,L}_{m3}^{n}{,L}_{m4}^{n}{,V}_{m5}^{n};n=1,2,\ldots ,k)$ denote a GPS tracking record of order *m*. $I_{m1}^{n}$, $T_{m2}^{n}$, $L_{m3}^{n}$, $L_{m4}^{n}$, and $V_{m5}^{n}$ denote the five data fields of the car sharing GPS tracking record *n*.

Then, the travel time and distance of each car sharing order can be calculated using the car sharing GPS trajectory data according to Equation (1) and Equation (2), respectively:

(1) $$T_{m}{=T}_{m2}^{k}{-T}_{m2}^{1}$$

(2) $$S_{m}=\overset{k-1}{\underset{i=1}{\sum }}\frac{{(T}_{m2}^{i+1}{-T}_{m2}^{i}{)(V}_{m5}^{i+1}{+V}_{m5}^{i})}{2}$$

Here, $T_{m2}^{1}$ and $T_{m2}^{k}$ denote the time stamp corresponding to the first tracking record and the time stamp corresponding to the last tracking record of the order *m*. $V_{m5}^{i}$ denotes the instantaneous speed corresponding to the tracking record *i* of the order *m*.

#### 3.3.3. Step 3: Matching the Administrative District and Functional Zone for Each Car Sharing Order

We categorize all the car sharing orders according to their corresponding administrative districts and functional zones. Firstly, for each car sharing order, we obtain the latitude and longitude coordinate of its pick-up station. Then, we use the Gaode LBS open platform [47] to perform reverse address resolution on the latitude and longitude coordinate, so as to obtain the administrative district and functional zone where the pick-up station is located. The administrative districts of Beijing are Dongcheng, Xicheng, Chaoyang, Haidian, Fengtai, Shijingshan, Changping, Shunyi, Tongzhou, Fangshan, Daxing, Miyun, Yanqing, Pinggu, Mentougou, and Huairou. According to the classification standard of POIs provided by Gaode LBS, Beijing has 19 functional zones, including functional zones of catering service, road furniture, names and addresses of places, tourist attractions, enterprises, shopping service, transportation service, financial and insurance service, culture and education service, motorcycle service, auto service, auto repair, car sales, commercial house, sports and recreation service, transit facilities, medical service, government organization and social group, accommodation service.

## 4. Methodology

In this section, we propose a comparative advantage model to quantify the travel-cost of travelers by using car sharing or taxis to travel, respectively. The travel-costs here consist of monetary expenses and non-monetary expenses [48]. The monetary expense refers to the direct cost for using vehicles, i.e., the fare paid for car sharing orders or taxi orders. The non-monetary expenses, i.e., time cost, includes the cost of time that travelers spend in finding a car and transferring from vehicles.

**Figure 3:**
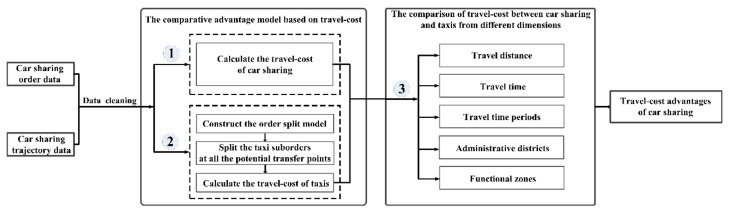
Methodology of the study.

Figure 3 shows the flow chart of the methodology. As shown in Figure 3, we then proceed in three phases after data cleaning. In the first phase, we use car sharing trajectory data to calculate the travel-cost of using car sharing based on the charge standard of car sharing and the average wage level of travelers. In the second phase, the same car sharing trajectory data is used to calculate the potential travel-cost of taking taxis to complete the same rides as car sharing. This phase contains three steps: First, we split each car sharing order into some taxi suborders at the stay points whose parking duration is more than 5 min; Second, the taxi suborders that still contain the stay points whose parking duration is more than 5 min are further split; Third, we calculate the travel-cost of each car sharing order in the scenario of taking taxis based on the taxi charge standard and the average wage level of travelers. In the third phase, we compare the travel-cost of car sharing and that of taxis from the dimensions such as travel distances, travel time, travel time periods, administrative districts and functional zones to figure out the travel-cost advantages of car sharing.

### 4.1. Setting Charge Standards

The fare of car sharing orders is charged based on both travel time and distance, which can be calculated as shown in Equation (3):

(3) $$F_{m-share}{=p}_{t}T_{m}{+p}_{s}S_{m}$$

where $F_{m-share}$ denotes the fare of a car sharing order *m*. $p_{t}$ and $p_{s}$ represent the travel fare per unit of time (t) and the travel fare per unit of distance (s), respectively. $T_{m}$ and $S_{m}$ denote the travel time and distance of a car sharing order *m*, respectively.

Table 3 shows the charge standard of car sharing in Beijing. Meanwhile, we also list the charge standard of taxi in Beijing, as shown in Table 4.

In the next part, we will calculate and compare travel-cost by using the transportation modes of both car sharing and taxis, respectively. The travel-cost includes the travel time cost and the fare paid for the orders. As far as the travel time cost, it could be the value generated by the opportunity cost of the time travelers spend on the journey [50]. We quantify the travelers’ travel time cost according to their average individual incomes [50].

According to the document issued by Beijing Municipal Human Resources and Social Security Bureau, the average monthly salary of each employee in Beijing in 2017 was 8467 yuan (i.e., about 1252 dollars). Based on this, the income C of each employee in Beijing in 2017 could be recorded as 0.88 yuan/min (i.e., about 0.13 dollars/min) in this paper.

### 4.2. Building Comparative Advantage Model Based on Travel-Cost

In this section, we construct a comparative advantage model based on travel-cost according to the charge standards of car sharing and taxi, in which car sharing GPS trajectory data are used to calculate the travel-cost of car sharing and that of taxis, so as to explore the competitive advantages of car sharing based on the travel-cost.

**Figure 4:**
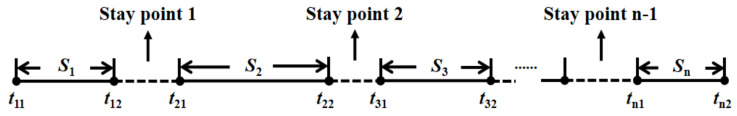
A car sharing order that contains *n* consecutive travel distances.

Let ${P=(S}_{1}{,S}_{2}{,S}_{3}{,\ldots ,S}_{n})$ denote *n* consecutive travel distances of a car sharing order, as shown in Figure 4, where S is a consecutive travel distance of a car sharing order. *n* is the number of consecutive travel distances of a car sharing order. $S_{l}(l=1,2,\ldots ,n)$ is the *l*-th consecutive travel distance. *t* denotes the starting time or the finishing time of a consecutive travel distance. $t_{l1}$ and $t_{l2}$ refer to the starting time and the finishing time of the *l*-th consecutive travel distance, respectively.

#### 4.2.1. Assumptions of the Model

We put forward five assumptions to facilitate computations as follows:

(1) We assume that travelers behave like “homo economicus”.
(2) According to the survey data [51], it is found that about 75% of the respondents can accept a distance between 0.5 km and 1 km when it comes to the accessibility of car sharing stations. Therefore, we assume that the distance L from the place of departure to the pick-up station of car sharing is 0.5 km.
(3) We assume that travelers either walk or ride bikes to the pick-up station of car sharing and their walking speed $v_{1}$ and cycling speed $v_{2}$ are 4.5 km/h and 15 km/h according to prior knowledge [33], respectively.
(4) Let Δt denote the time cost of travelers searching and waiting for a new taxi, which is supposed to be 5 min in our research subjectively.
(5) We do not distinguish the gap in driving experience between taxi drivers and car sharing drivers for those regular car sharing drivers can use the onboard navigation system when they are not familiar with the travel routes, which can help them choose the optimum travel route (shortest or congestion-avoiding one). Thus, we assume that both the car sharing drivers and taxi drivers would choose the most efficient routes when they are driving. In other words, they would choose the same routes for the same travel in our research.

#### 4.2.2. The Comparative Advantage Model Based on Travel-Cost

The comparative advantage model based on travel-cost includes two parts: using the GPS trajectory data of car sharing orders to calculate the travel-cost of car sharing and that of taxis. Firstly, we use car sharing GPS trajectory data to calculate the travel-cost of using car sharing, including (1) the time cost for travelers to get to the pick-up station (on foot or by bike) and (2) the monetary cost spending in driving from the pick-up station to the drop-off station by car sharing. The specific calculation method is shown in Equation (4):

(4) $$F_{m}=\left\{\begin{array}{c} C\frac{L}{v_{1}}{+F}_{m-\mathrm{share}}\;\mathrm{walking}\;\mathrm{to}\;\mathrm{the}\;\mathrm{pick}-\mathrm{up}\;\mathrm{station}\\ C\frac{L}{v_{2}}{+F}_{m-\mathrm{share}}\;\mathrm{cycling}\;\mathrm{to}\;\mathrm{the}\;\mathrm{pick}-\mathrm{up}\;\mathrm{station} \end{array}\right.$$

where $F_{m}$ refers to the travel-cost of a car sharing order m. C refers to the revenue per unit time of each traveler. $\frac{L}{v_{1}}$ is the time that travelers spend in walking to the pick-up station. $\frac{L}{v_{2}}$ is the time that travelers spend in cycling to the pick-up station. $F_{m-\mathrm{share}}$ denotes the monetary cost (i.e., the fare of a car sharing order calculated according to Equation (3)) of traveling by car sharing.

Next, the same GPS trajectory data of car sharing orders are used to calculate the travel-cost in the case of taking taxis. The specific process of calculation is as follows:

Step 1: Construct the Order Split Model.

From the GPS trajectory data of car sharing orders, we find that there are many stay points in a trip from the pick-up station to the drop-off station. The stay point between two adjacent travel distances refers to a set of consecutive tracking records whose instantaneous speed is zero. Table 5 shows the parking durations of stay points and their frequency distribution. As shown in Table 5, the parking duration of most stay points is from 0 to 5 min, accounting for 91.84% of the total stay points.

When we calculate the potential travel-cost by taxis for the same ride by car sharing, we shall judge whether it is necessary to transfer (i.e., by ordering another taxi) at a stay point and where. This is because there is a low speed fee in the charge standard of taxi and travelers might stay a long time to do some tasks. In this case, the travelers would like to let the previous taxi go and order another one when needed. In general, along with the parking duration becoming longer, people would tend to transfer at a stay point. In this research, a stay point whose parking duration lasts more than 5 min would be supposed to be a potential transfer location. Thus, we should split the whole ride into two taxi rides at the stay point when calculating the travel-cost.

**Figure 5:**
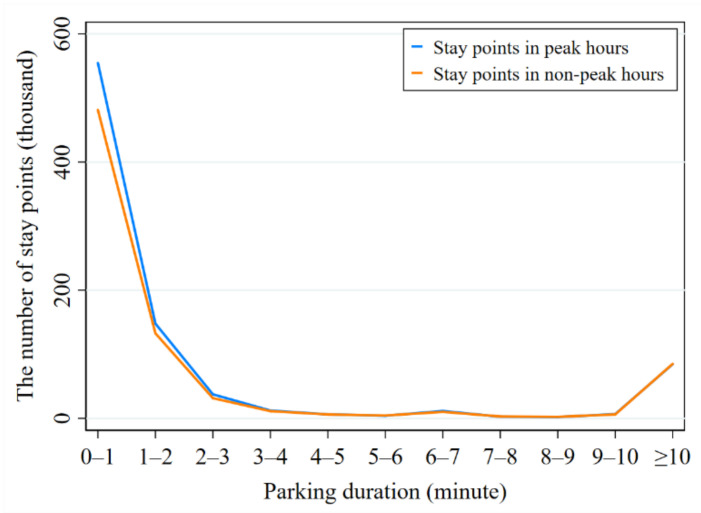
The quantitative distribution of stay points in different parking durations.

Figure 5 shows the quantitative distribution of stay points in peak hours (from 7:00 to 10:00 and from 17:00 to 20:00) and non-peak hours (from 10:00 to 16:00). From Figure 5, we can find that there is a certain difference between the number of stay points in peak hours and that in non-peak hours when the parking duration is less than 5 min. When the parking duration becomes more than 5 min, the number of stay points in peak hours tends to be consistent with that in non-peak hours, which indicates that traffic congestion would not lead to more stay points for vehicles. In other words, most stay points whose parking duration is more than 5 min are real stay points and they are not caused by the traffic congestion.

Considering that those stay points whose parking duration is more than 5 min might be potential transfer points for travelers. We need to judge whether it is necessary to transfer at these stay points by calculating and comparing the travel-cost between transferring to another taxi and not. Let *S_i_* denote the *i*-th consecutive travel distance after the stay point of *i*-1 in a car sharing order, *F*_1_ and *F*_2_ denote the travel-cost of transferring to another taxi and that of not. The terms *F*_1_ and *F*_2_ can be calculated by Equation (5) below:

(5) $$\left\{\begin{array}{c} F_{1}=C\Delta {t+F}_{S_{i}}\\ F_{2}{=F}_{low-speed}{+F}_{S_{i}}^{\prime } \end{array}\right.$$

where CΔt represents the time cost for travelers to transfer to another taxi (searching and waiting for another taxi). $F_{S_{i}}$ denotes the taxi fare of $S_{i}$ which is calculated according to the charge standard of taxi. $F_{S_{i}}^{\prime }$ also denotes the taxi fare of $S_{i}$, but it is influenced by $S_{1}$ to $S_{i-1}$ for the traveler use the same taxi to ride $S_{i-1}$ and $S_{i}$.

**Figure 6:**
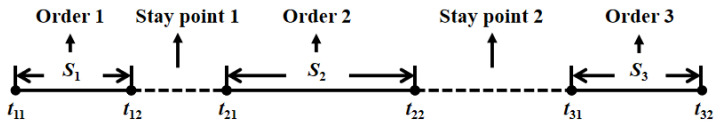
A car sharing order that has been split into three taxi suborders.

At each stay point of a car sharing order, if $F_{1}$ ≥ $F_{2}$, the ride would not be split at the current stay point. Otherwise, it would be split at the current stay point. Taking a car sharing order that has two stay points for example, if all the two stay points meet the split condition, the car sharing order would be split into three taxi suborders, as shown in Figure 6.

Step 2: Split the Taxi Suborders at all the Potential Transfer Points.

**Figure 7:**
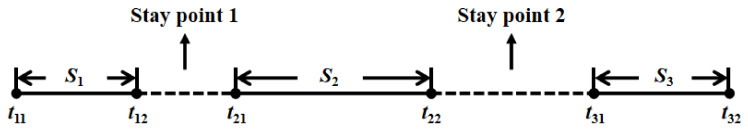
A taxi suborder that has two stay points (or possible transfer points) whose parking duration is more than 5 min.

A car sharing order would not be split at the stay points which do not meet the split conditions. However, those stay points may also be transfer points in whole. In the next, we judge whether the taxi suborders that contain the stay points whose parking duration is more than 5 min would be split. We take a taxi suborder that has two stay points (or possible transfer points) as an example, as shown in Figure 7. The following three cases should be considered when we discuss whether this taxi suborder should be split and how to be split.

(1) Travelers do not transfer to another taxi at any of these two stay points. In this case, travelers take the same taxi to complete the whole trip $S_{1}{+S}_{2}{+S}_{3}$, and we record the travel-cost of the whole trip as $G_{1}$.
(2) Travelers transfer at one of the two stay points. If travelers transfer to another taxi at the stay point 1 and do not transfer at the stay point 2, that is, travelers take two different taxis to complete the trip $S_{1}$ and the trip $S_{2}{+S}_{3}$ respectively. We record the total travel-cost of these two trips as $G_{2}$. Conversely, if travelers choose to transfer to another taxi at the stay point 2 rather than at the stay point 1, that is, travelers take two different taxis to complete the trip $S_{1}{+S}_{2}$ and the trip $S_{3}$ respectively. We record the total travel-cost of these two trips as $G_{3}$.
(3) Travelers transfer at both two stay points. In other words, travelers take three different taxis to complete the trips $S_{1}$, $S_{2}$, and $S_{3}$, respectively. We record the total travel-cost of these three trips as $G_{4}$.

Then, by comparing $G_{1}$, $G_{2}$, $G_{3}$ and $G_{4}$, we decide whether this taxi suborder should be split and how to be split. For example, if $G_{4}$ is the least, this taxi suborder will be split at the stay points 1 and 2.

Step 3: Calculate the Travel-Cost.

**Figure 8:**
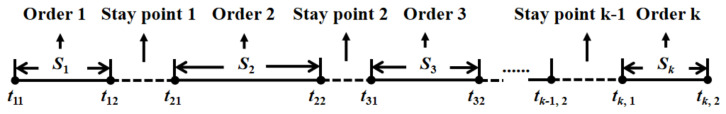
A car sharing order that has been split into k taxi suborders.

After step 1 and 2, each car sharing order will be split into many taxi suborders. Taking a car sharing order that has been split into k taxi suborders $D_{i}(i=1,2,\ldots ,k)$ as an example, as shown in Figure 8. The travel-cost $F_{D_{i}}$ of each taxi suborder $D_{i}$ is calculated according to the taxi charge standard in Table 4. The travel-cost $F_{taxi}$ of each car sharing order in the scenario of taking taxis can be calculated by Equation (6):

(6) $$F_{taxi}=\left(k-1\right)C\Delta t+\overset{k}{\underset{i=1}{\sum }}F_{D_{i}}$$

where CΔt represents the time cost for travelers to transfer to another taxi (searching and waiting for another taxi).

## 5. Results and Analysis

### 5.1. The statistical Characteristics of Car Sharing Orders and Tracking Records

**Figure 9:**
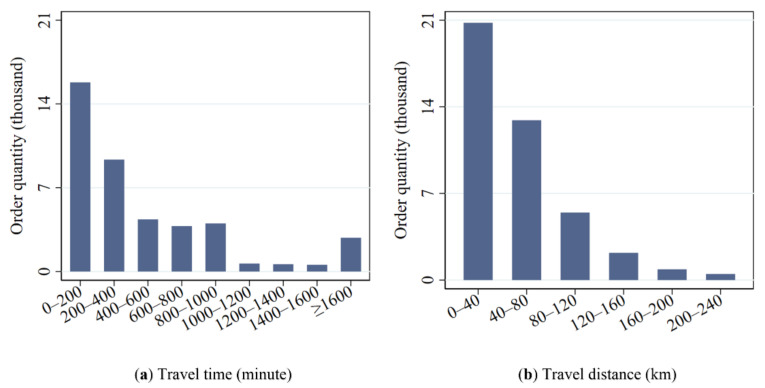
Quantitative distribution of car sharing orders over different travel time (**a**) and travel distances (**b**).

Figure 9 shows the quantitative distribution of car sharing orders as per different travel time (a) and travel distances (b) (the travel time refers to the overall car rental time which contains the parking duration and consecutive driving time; some users may rent a shared car for several days before returning it. Therefore, the travel time and distance of some car sharing orders are very long). It can be seen from the figure that the number of car sharing orders shows a decreasing trend with the increase of travel time and travel distance. The number of car sharing orders with a travel time of less than 1000 min (about 16.67 h) accounts for 88.88% of the total; and the average travel time of these orders whose travel time is less than 1000 min is 340 min (about 5.67 h). The number of car sharing orders with a travel distance less than 120 km accounts for 91.68% of the total; and the average travel distance of these orders whose travel distance is less than 120 km is 43 km (this is because these car sharing orders might contain several continued driving rides and they are not just used for commuting. For example, an individual might go to the suburbs for pleasure and then go back the next day. In this case, the travel distance of this order might be a hundred kilometers or more).

**Figure 10:**
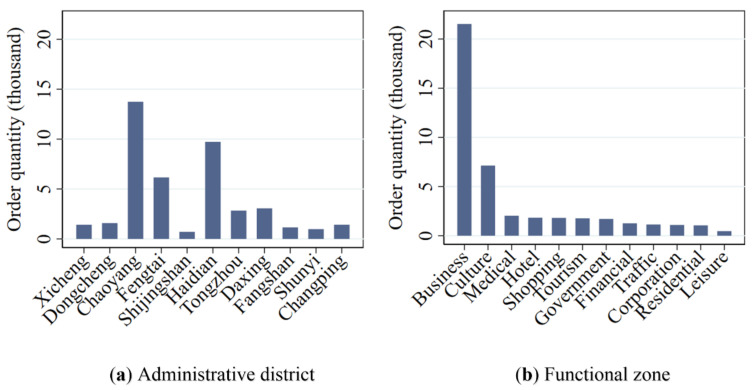
Quantitative distribution of car sharing orders in each administrative district (**a**) and functional zone (**b**). Notes: The administrative districts in Figure 10a are ranked in an ascending order by their distance from the city center.

Figure 10 shows the quantitative distribution of car sharing orders in different administrative districts (a) and functional zones (b). As can be seen from Figure 10a, about 83% of total car sharing orders come from Chaoyang, Haidian, Fengtai, Daxing and Tongzhou. The reasons for this can be summarized in two main aspects: First, the car sharing stations are mainly distributed in these administrative districts. Second, these five administrative districts gather a large number of commercial places, universities and tourist attractions, and have a relatively perfect transportation infrastructure. These areas are densely-populated and play an important role in people’s residence, life and work. People who live here have a huge demand for travel. Therefore, shared cars in these five districts have more chances to be used compared with the other administrative districts.

In addition, it can be observed from Figure 10b that car sharing orders are mainly distributed in the functional zones of business and culture, accounting for 67% of the total orders. This is because there are a lot of industrial areas and commercial buildings in the functional zone of business and a large number of media organizations, museums, libraries and planetarium in the functional zone of culture. Most of the people who appear in these places are young and well-educated. They are more open to new things and environmentally conscious [25]. Therefore, shared cars are used more often in these functional zones.

**Figure 11:**
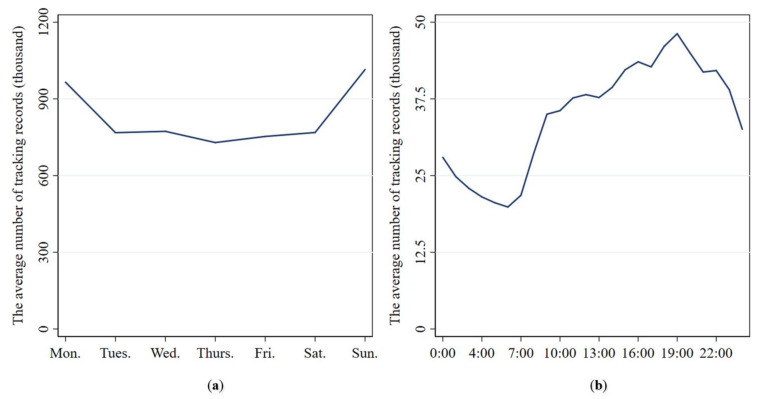
Quantitative distribution of car sharing tracking records in a week (**a**) and in different time periods (**b**).

Figure 11 shows the quantity distribution of car sharing tracking records in a week (a) and in different time periods (b). On the whole, it can be seen from Figure 11a that the average number of car sharing tracking records is highest on Sunday, followed by Monday. The average number of tracking records is evenly distributed from Tuesday to Saturday. This indicates that travelers use car sharing more often on Monday and Sunday. Namely, the usage frequencies of car sharing are relatively higher on Monday and Sunday.

As can be seen from Figure 11b, the average number of car sharing tracking records has obvious peaks and troughs during the day. The average number of tracking records gradually increases from 5:00 to 19:00, while it shows a downward trend from 19:00 to 24:00 and from 0:00 to 5:00. This pertains to the fact that people’s main travel purpose from 5:00 to 9:00 is commuting. Thus, the demand for car sharing is relatively concentrated during that time period. From 9:00 to 19:00, people have more varied travel purposes and their departure times are relatively dispersed. In the other two time periods (from 19:00 to 24:00 and from 0:00 to 5:00), most people choose to rest at home and the demand for travelling is relatively small.

### 5.2. Competitive Advantage of Car Sharing in Ddifferent Travel Distances

**Figure 12:**
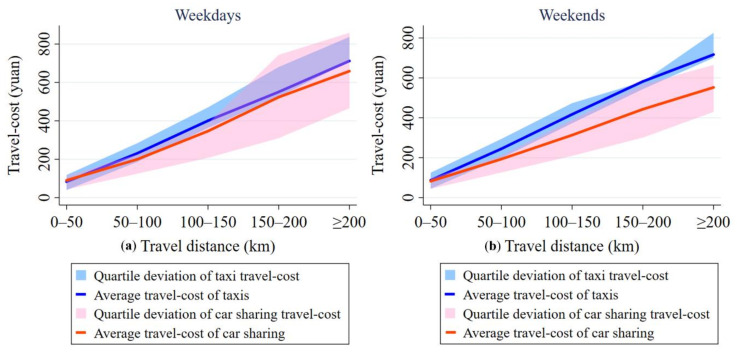
Comparison of travel-cost between taxis and car sharing in different travel distances on weekdays (**a**) and on weekends (**b**).

Figure 12 shows the distribution of the travel-cost of car sharing and taxis in different travel distances. From Figure 12, we can find that the travel-cost of shared cars is not far different from that of taxis in terms of short-distance trips on both weekdays and weekends. However, for middle- and long-distance travel, the travel-cost of taxis is significantly higher than that of shared cars (when the travel distance is more than 50 km, the statistical significance (*p*-values = 0) indicates that we should reject the null hypothesis H0 at the significance level of 0.05 by using T-test (H0: The travel-cost of taxis is less than that of car sharing). The results of the single sample T-test and the paired-samples T-test are shown in Table A1 and Table A2 in the Appendix A, respectively). For example, the difference in average travel-cost between shared cars and taxis is minor when the travel distance is within 50 km, but the average travel-cost of traveling by taxi is far higher than that of traveling by shared cars in the trips over a distance of 100 km; the average travel-cost of taxis is 10% higher than that of car sharing on weekdays, whereas on weekends, the average travel-cost of taxis is 30% higher than that of car sharing.

We can also find some differences between weekdays and weekends from Figure 12. For example, compared with weekdays, it is more cost-effective to travel by shared cars rather than taxi for short-distance trips on weekends only. Meanwhile, for middle- and long-distance travel, the average travel-cost for travelers to use car sharing is less than that of taking taxis both on weekdays and on weekends. Specifically, in the case of the travel within 50 km, the average travel-cost for taking taxis is 5 yuan (i.e., about 0.74 dollars) higher than that for traveling by shared cars on weekends, while it is 8 yuan (i.e., about 1.18 dollars) lower on weekdays. As for the travel with 50 to100 km, the average travel-cost of taxis is 51 yuan (i.e., about 7.54 dollars) higher than that of shared cars on weekends; this gap in the travel-cost between the two travel modes is 60% higher than that on weekdays. Besides, the average travel-cost of taking taxis is 116 yuan (i.e., about 17.15 dollars) higher than that of shared cars on weekends in trips over 100 km; and this gap in the average travel-cost between the two travel modes is 140% higher than that on weekdays.

On the whole, we find that the travel-cost advantage of taking taxis is relatively higher than for using car sharing on weekdays only, when it comes to short-distance travel. Meanwhile, compared with taking taxis, the travel-cost advantage of taking car sharing is relatively higher on weekends than on weekdays in terms of middle- and long-distance travel. Because people generally travel for a single purpose and travel mainly for the activities regarding work, study, leisure and entertainment, and shopping in short-distance travel. In this case, travelers often give priority to traveling by taxis, due to full-time operation, which means that they can provide fast, flexible, and around-the-clock service; by contrast, car sharing is less convenient than taxis, for travelers have to get to the pick-up and drop-off stations. Another reason pertains to the fact that travelers may either go to more than one destination or go touring in a middle- or long-distance trip, which means that if they choose to take taxis, they will face a range of inconveniences such as transferring from and waiting for vehicles. Moreover, travel-cost of taking taxis is higher than car sharing, resulting from the long travel distances and the different charge standards. Therefore, travelers prefer to take shared cars when it comes to the middle- and long-distance travel, as car sharing is convenient, comfortable, and relatively inexpensive.

### 5.3. Competitive Advantage of Car Saring in Different Travel Time

**Figure 13:**
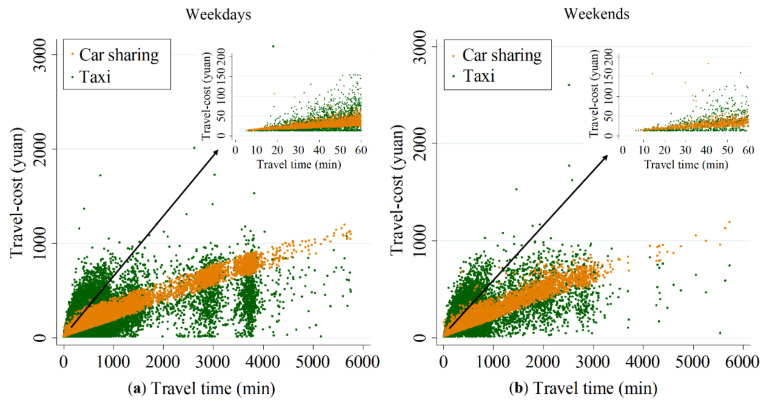
Comparison of travel-cost between taxis and car sharing in different travel time on weekdays (**a**) and on weekends (**b**).

Figure 13 shows the travel-cost distribution of car sharing and taxis in different travel time on weekdays and on weekends. Overall, when the travel time is too short, the travel-cost of taking car sharing is a little higher than that of taking taxis both on weekdays and weekends. As the travel time increases, the travel-cost of taxis becomes gradually much higher than that of car sharing (when the travel time is from 20 to 60 min, the statistical significance (*p*-values = 0) indicates that we should reject the null hypothesis H0 at the significance level of 0.05 by using T-test (H0: The travel-cost of taxis is less than that of car sharing). The results of the single sample T-test and the paired-samples T-test are shown in Table A3 and Table A4 in the Appendix A, respectively).

Specifically, the average travel-cost of car sharing is 2 yuan (i.e., about 0.30 dollars) higher than that of taxis on weekdays and 4 yuan (i.e., about 1.45 dollars) higher than that of taxis on weekends when the travel time is within 20 min. When the travel time is from 20 to 60 min, the average travel-cost of taxis starts to become higher than the travel-cost of shared cars. As the travel time increases gradually, especially when it increases to more than 1000 min (roughly more than 16 h), the travel-cost of shared cars becomes much higher than that of taxis, with the average travel-cost of car sharing being approximately 30% higher than that of using taxis on weekdays and 10% higher than the average travel-cost of using taxis on weekends.

We can also find some differences between weekdays and weekends from Figure 13. For example, the travel-cost advantage of traveling by taxi on weekdays is greater than it is on weekends when the travel time is either very short (less than 20 min) or very long (more than 1000 min) (this is because there might be a lot of stay points in a car sharing order. A car sharing order could be split into many taxi suborders. In this situation, the continuous driving time of each taxi suborder would be far less than the travel time of this car sharing order. In other words, a car sharing ride whose travel time is very long may be done by many taxis). For instance, the average travel-cost of travelling by shared cars is approximately 132 yuan (i.e., about 19.51 dollars) more than that of travelling by taxi when the travel time is more than 1000 min. Such a gap in the average travel-cost between shared cars and taxis on weekdays is approximately 150% higher than it is on weekends. Besides, there is marginal difference in the travel-cost between shared cars and taxis in other travel time both on weekdays and on weekends.

In addition, as can be seen from Figure 13, the variance of the travel-cost of car sharing is smaller than that of taxis across the travel time. On one hand, this is because the pricing standard of car sharing is relatively simple, which is only charged by travel time and distance. When the travel time is fixed, the variance of travel-cost of car sharing is mainly caused by the travel distance. However, due to the influence of road’s speed limits and battery range, the travel distance of car sharing would always fluctuate within a range when the travel time is fixed. On the other hand, the travel-cost of taxis is calculated based on the car sharing orders. The situation varies from order to order when the travel time is the same. For example, some car sharing orders have a longer parking time which would be seen as people’s transfer time of taxi and has no charge. However, some orders do not have such a long parking time, so their travel-cost of taxis is relatively higher. Therefore, the variance of travel-cost of taxis is bigger. Besides, we can also find that the variance of travel-cost of car sharing increases with the increase of travel time. For example, from the internal chart of Figure 13 we can find that the variance of travel-cost of car sharing increases significantly with the increase of travel time when the travel time is within 60 min. This is because the variance of travel-cost of car sharing is caused by travel distance when the travel time is fixed. Generally, with the increase of travel time, the travel distance would increase as well. Therefore, the part of travel-cost charged by travel distance would get larger and the variance of travel-cost would get bigger.

On the whole, it is more economical for travelers to travel by taxi than by shared cars when the travel time is either more than 1000 min or less than 20 min. In other travel times, traveling by car sharing has a greater travel-cost advantage for travelers than traveling by taxi, because short travel time usually means the short travel distance and the relatively simple travel process. In this case, travelers are more likely to take a taxi directly from the place of departure to complete the trip, rather than go to the nearby car sharing outlets to pick up shared cars first. Conversely, long travel time corresponds to long travel distances and various travel purposes. For such a case, travelers tend to take taxis to complete their travel due to parking problems and high parking fees. From the perspective of travel-cost, the charge standard of car sharing means that it is relatively cheap to travel by shared cars, rendering travelers to use car sharing in other travel time, as car sharing provides convenient, comfortable and inexpensive services.

### 5.4. Competitive Advantage of Car Sharing in Different Travel Time Periods

**Figure 14:**
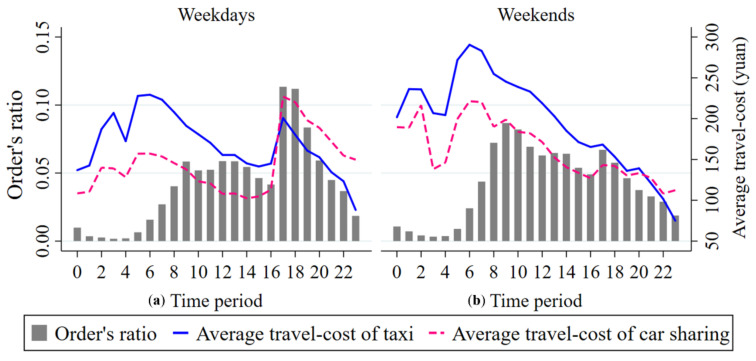
Comparison of travel-cost between taxis and car sharing in different time periods on weekdays (**a**) and on weekends (**b**).

Figure 14 shows daily changes in the proportion of car sharing orders and in the average travel-cost of car sharing and taxis. It can be seen from Figure 14 that the daily change in the average travel-cost of car sharing and that of taxis present consistent trends both on weekdays and on weekends, while the average travel-cost of taking shared cars and that of taking taxis are significantly different.

Specifically, the average travel-cost of traveling by taxi is higher than it is by car sharing during the hour periods before 21:00 on weekends, while traveling by car sharing is much more expensive than traveling by taxi after 17:00 on weekdays. In addition, the average travel-cost of car sharing and that of taxis both clearly present double rush hour periods from 5:00 to 6:00 and from 17:00 to 18:00 on weekdays. However, this is not the case for weekends, as the average travel-cost of car sharing and that of taxis both reach the peak only from 6:00 to 7:00. This is because on weekdays travelers mainly commute to work, with constrained commuting time. Meanwhile, travelers need to leave home earlier than rush hours in the morning and leave work later than rush hours in the evening due to job-housing separation to avoid the inconvenience caused by road congestion. On the other hand, residents are mainly engaged within leisure places such as tourist attractions, catering locations and outdoor shopping malls on weekends, as journeys for such areas are random and decentralized from the perspective of departure time, so a sharp decline both in the average travel-cost of car sharing and taxis appears after rush hours on weekends’ morning.

It can also be observed from Figure 14 that the average travel-cost for traveling by car sharing and for traveling by taxi both show an upward variation trend from 0:00 to 5:00 on weekdays but a downward variation trend at that time period on weekends, as residents travel mainly for commuting on weekdays. Due to the fact that more people tend to either pick up a shared car or take a taxi before rush hours in the morning, their demand for both shared cars and taxis also increases. On weekends, most people are still staying at home for rest from 0:00 to 5:00, so that people’s demand for both car sharing and taxis is low. After 6:00, the travel-cost of both travel modes falls rapidly. Moreover, we also find that there is a definite correlation between travel-cost and order volume for both car sharing and taxis. For example, on weekdays, the average travel-cost of taking shared cars and that of taking taxis increase gradually from 0:00 to 5:00, whereas the order quantity decreases, indicating a negative correlation between travel-cost and order volume; the correlation coefficients are −0.9 (taxi) and −0.7 (car sharing). Besides, the average travel-cost for both shared cars and taxis and the number of car sharing orders both decrease gradually from 17:00 to 24:00 on weekdays. In this case, there is a positive correlation between travel-cost and order volume and the correlation coefficients are 0.964 (taxi) and 0.986 (car sharing).

On the whole, traveling by shared cars is more economical than traveling by taxis before rush hours on weekdays’ evening, while there is a greater travel-cost advantage with taking taxis after the evening peak. On weekends, the travel-cost advantage for travelers to travel by taxi is greater after 21:00, whereas the travel-cost advantage of taking car sharing is bigger than taking taxis during other time periods. On one hand, this is because travel on weekdays is done mainly for commuting. Travel demand is relatively higher during the commuting time. In this case, it will be difficult to hail taxis for travelers who choose to travel by taxi. On weekends, travelers travel mainly for the purposes of leisure and entertainment, which means that they have a higher demand for comfortable, flexible, and convenient traffic modes. Compared with taxis, car sharing is in line with people’s psychological expectations in those respects. On the other hand, the travel distance and the travel time are relatively shorter and the travel process is relatively simple when residents travel at night. In this case, travelers often prefer to take taxis directly for their travel purposes considering self-security and the time cost for picking up and dropping off shared cars.

### 5.5. Competitive Advantage of Car Sharing in Different Districts

**Figure 15:**
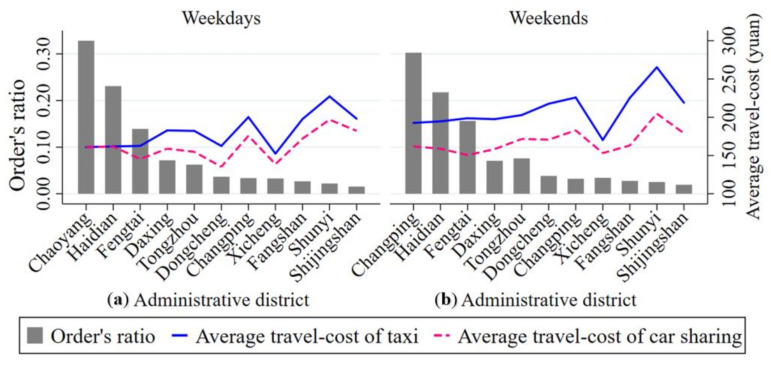
Comparison of travel-cost between taxis and car sharing for each administrative district on weekdays (**a**) and on weekends (**b**).

We first analyze the distribution of both car sharing order volume and the travel-cost for taking car sharing and taxis in each administrative district. As Figure 15 indicates, the regional distribution of car sharing in Beijing is extremely imbalanced and varies a lot in different administrative districts from the perspective of car sharing order volume. More specifically, there are more car sharing orders in Chaoyang, Haidian and Fengtai districts. The proportions of car sharing orders in these three administrative districts on weekdays and weekends are 70% and 68%, respectively. From the perspective of the travel-cost, the average travel-cost of car sharing in each administrative district is lower than that of traveling by taxi both on weekdays and on weekends. The difference in the average travel-costs between the two travel modes is much more significant on weekends than it is on weekdays. For example, Chaoyang and Haidian districts both present marginal differences in the average travel-cost for taking car sharing and taxis on weekdays. By contrast, the average travel-cost of taking taxis to travel is 20% higher that of taking car sharing in both Chaoyang and Haidian districts on weekends.

It can also be observed from Figure 15 that people are more likely to use car sharing to travel on weekdays than on weekends. For instance, the order volume on weekdays is 180% more than that on weekends in Chaoyang district. Moreover, from the perspective of travel-cost, the travel-cost advantage of traveling by car sharing is greater on weekends in the main urban area (Dongcheng, Xicheng, Chaoyang, Haidian, Fengtai and Shijingshan districts) and in the suburbs. Specifically, the average travel-cost of taking taxis is approximately 36 yuan (i.e., about 5.32 dollars) higher than that of taking car sharing in the main urban area, which is 600% higher than on weekdays. In the suburbs, the average travel-cost of traveling by taxi is approximately 42 yuan (i.e., about 6.21 dollars) higher than that of traveling by car sharing on weekends, which is 60% higher that on weekdays. In conclusion, the travel-cost advantage of traveling by car sharing is greater on weekends than on weekdays not only in the main urban area but also in the suburbs, which is mainly attributable to the following reasons. First, residents travel mainly for leisure and recreational activities on weekends compared with weekdays. In this case, their demand for comfort and flexibility while traveling is higher on weekends. Second, the service characteristics of car sharing well cater to residents’ demands for a comfortable and flexible trip, which makes car sharing have a higher travel-cost advantage on weekends.

**Figure 16:**
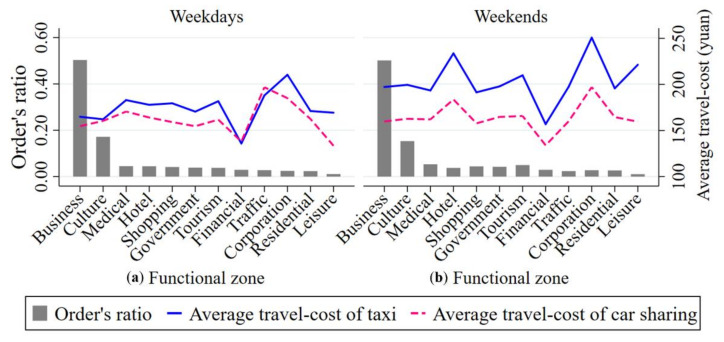
Comparison of travel-cost between taxis and car sharing for each functional zone on weekdays (**a**) and on weekends (**b**).

We next analyze the distribution of both car sharing order volume and the travel-cost of taking car sharing and taxis in each functional zone. As shown in Figure 16, the distribution of car sharing among functional zones is quite different in the light of car sharing order volume. For example, car sharing orders are mainly distributed in functional zones of business and culture which account for 68% of the total number of car sharing orders on weekdays and 65% on weekends. From the perspective of travel-cost, traveling by shared cars has a bigger travel-cost advantage for travelers than it is by taxi on both weekdays and weekends in each functional zone. For instance, the average travel-cost of taking car sharing to travel is 1.7 yuan (i.e., about 0.25 dollars) lower than that of taking taxis on weekdays and 36.8 yuan (i.e., about 5.44 dollars) lower on weekends.

Figure 16 also shows that the utilization rate of shared cars is higher on weekdays than that on weekends. For instance, the volume of car sharing orders on weekdays is roughly 160% more than that on weekends in the functional zone of business. In addition, taking car sharing to travel has a higher travel-cost advantage on weekends. Taking the functional zone of culture as an example, the average travel-cost of traveling by taxi on weekends is 36.8 yuan (i.e., about 5.44 dollars) higher than it is by car sharing. This gap in the average travel-cost between taking taxis to travel and taking car sharing to travel on weekends is roughly 2100% higher than that on weekdays. Moreover, from Figure 16, we can also find that the top three functional zones with the greatest differences in the travel-cost advantage of car sharing between weekdays and weekends are the function zones of culture, financial and traffic.

## 6. Discussion

### 6.1. The Comparison with Previous Literature

In this paper, we figured out the travel-cost advantages of using car sharing versus taking taxis, from different dimensions such as travel distances, travel time, travel time periods, administrative districts and functional zones. In this process, the biggest challenge is that we need to split each car sharing order into many comparable taxi suborders for there are a lot of stay points in each car sharing ride, which do not conform to the characteristics of a taxi ride. Thus, we constructed a comparative advantage model based on travel-cost to split each car sharing order and measure its travel-cost and potential travel-cost of taking taxis. The model proved to be quite a reasonable and effective method, which provided insights into the travel-cost difference between the two travel modes. Compared with previous similar studies [25,26,45], our research mainly contributes to the following three aspects:

Firstly, we used the car sharing orders and their trajectory data to figure out their distributions in different travel distances, travel time, travel time periods and in different areas, which reflects the demand of travelers for traveling by car sharing. Differently to similar studies that have only focused on single dimension of car sharing users’ travel characteristics [25,26], we studied the travel patterns and travel characteristics of car sharing users in a more comprehensive and detailed way.

Secondly, we not only calculated the monetary cost of car sharing orders, but also considered and quantified the time cost of travelers spending in getting to the pick-up station of car sharing. In the process of quantifying the time cost, we took into consideration the average wage level of employees in Beijing and converted the time cost into the income.

Thirdly, for each car sharing order, we proposed a comparative model to calculate its potential travel-cost by taxi. We found that there are a large number of stay points in a car sharing order, which is different from traveling by taxi. Therefore, we provide a method to split each car sharing order into comparable taxi suborders according to the least-cost principle, which is different from Hui et al. [45] who split each car sharing ride at the stay points whose parking duration is more than 720 s without considering travelers’ transfer choices.

Our research comes to some similar conclusions to previous studies. For example, Dias et al. [25] found that the well-educated residents and those with higher incomes in the areas with great population density tend to use car sharing mode to travel. In our research, we found that about 83% of total car sharing orders in Beijing come from Chaoyang, Haidian, Fengtai, Daxing and Tongzhou which are densely-populated and play an important role in people’s residence, life and work. In addition, we found that car sharing has travel-cost advantages on long-distance travel and its travel-cost advantages are relatively larger on weekends. The market competition between car sharing and taxis is fiercer in the central areas. These findings reflect a similar view to Hui et al. [45].

### 6.2. The Relationships Between Car Sharing and Other Modes of Transportation

Based on a study that explores the impacts of car sharing on the urban transportation system, it can be found that car sharing has both a complementary and competitive role in other transport modes such as bus, subway, bike sharing, taxi and private car [52]. Specifically, there exists in more of a complementary relationship between car sharing and bus, subway, bike sharing [52]. For example, most buses and subways are out of service at night, while car sharing can provide round-the-clock service, which makes up for the shortage of buses and subways in terms of the operational time at night. In addition, car sharing also can be deployed in areas where are lacking public transportation lines to meet people’s travel needs in these areas. What’s more, the transport connection between car sharing and bike sharing can provide travelers with higher accessibility to destinations and a relatively free and comfortable travel experience. There is also some market competition between car sharing and bus or subway. For example, car sharing as well as bus and subway can meet people’s demand for medium- and long-distance travel. When people are more sensitive to the cost than to the comfort and privacy, bus and subway are preferred.

However, there are obvious competitive relationships between car sharing and private car, taxi [52]. On one hand, car sharing has a high potential to reduce people’s demand for the purchase or usage of private cars [53,54]. On the other hand, user groups of car sharing and taxis have a high degree of coincidence. However, unlike taxis, car sharing is only available to travelers who own a driving license and is still in the early stage of development. Therefore, taxis are more widely used than car sharing. In addition to the competitive relationship, it is worth mentioning that car sharing complements private cars and taxis in some cases. For instance, people could use car sharing when they cannot hail a taxi or the use of private cars is restricted on certain days.

In consideration of the high similarity between car sharing and taxis, we focus on the competitive relationship between car sharing and taxis. Our work would be beneficial to improve the reasonable positioning of car sharing in the urban transportation system, provide decision support for the coordinated development of car sharing and other transportation modes to make people’s travel more convenient.

### 6.3. The Comparison Between Car Sharing and Taxis

Other than the analyses on travel-cost mentioned above, this subsection gives a brief comparison between car sharing and taxis from other aspects:

First, in terms of the pricing mechanism, taxis’ pricing mechanism is more diversified, taking into account various situations such as the low-speed fee, the empty-cruise fee and the night-time charge. In contrast, car sharing is mainly charged by the travel time and travel distance. The charge standard of car sharing is mostly the same at different time periods. In addition, car sharing reduces the cost of specialized drivers through users’ self-driving. Therefore, car sharing has a certain price advantage compared with taxis.

Second, as far as the operating time is concerned, both car sharing and taxis are available 24 h a day. As far as the business pattern is concerned, car sharing and traditional taxis are both asset-heavy industries, as the vehicles must be operated and maintained by a professional team. The other modes of taxis such as online taxi-hailing and carpooling belong to an asset-light taxi mode.

Third, there is a certain gap between car sharing and taxis in terms of user groups. Car sharing has a smaller number of users than taxis. On one hand, this is because car sharing is still in the early stages of development. The imperfect legal system of car sharing and the unclear rights and liabilities in traffic accidents make an adverse impact on travelers’ driving experience [32]. On the other hand, supporting facilities such as stations and charging piles are imperfect, making car sharing can only cater to the travel demands of a small number of people [32]. Additionally, car sharing is only applicable to people who own a driving license. The driving skill is also a great threshold for users. People with disabilities and people without a driving license cannot use car sharing [52].

## 7. Conclusions

Understanding the competitive advantages of car sharing from the perspective of travel-cost is of great theoretical and practical significance to help relevant departments both improve the management framework for car sharing development and promote the fine planning level of urban shared transportation. In this paper, firstly, we use the GPS trajectory data of car sharing orders to construct a comparative advantage model based on travel-cost. Then, taking Beijing as an example, the travel-cost of car sharing and taxis is measured and analyzed, so as to explore the cost advantages of car sharing from the travel-cost’s perspective. A series of valuable findings obtained from our study are as follows:

First of all, it is more economical to travel by taxi than by car sharing in terms of short-distance travel both on weekdays and on weekends. Meanwhile, the travel-cost advantage for travelers to travel by car sharing is much greater on weekends for middle- and long-distance travel than that on weekdays. Furthermore, as the travel distance increases, the travel-cost advantage of traveling by car sharing on weekends also increases gradually. From the perspective of travel time, the travel-cost advantage of traveling by taxi is relatively high when the travel time is either too long or too short, whereas the travel-cost advantage of traveling by car sharing is relatively high in other travel time.

Secondly, the daily changes of the average travel-cost of car sharing and taxis present a consistent trend, with double peaks (5:00–6:00 and 17:00–18:00) on weekdays and one peak (6:00-7:00) on weekends. However, the difference in the average travel-cost between the two travel modes is significant. Specifically, on weekdays, the travel-cost advantage of using car sharing is much greater before rush hours in the evening, whereas there is a greater travel-cost advantage in taking taxis after rush hours in the evening. On weekends, it is more cost-effective for travelers to travel by taxi than to travel by car sharing after 21:00, while using car sharing has a greater travel-cost advantage at other time periods.

Finally, the regional distribution of car sharing is significantly imbalanced from the perspective of car sharing order volume, and the distribution of car sharing varies a lot among administrative districts and functional zones. From the perspective of the travel-cost, traveling by car sharing has a greater cost advantage on weekends than that on weekdays not only in the main urban area (Dongcheng, Xicheng, Chaoyang, Haidian, Fengtai and Shijingshan districts) but also in the remote suburbs. For functional zones, traveling by car sharing has a bigger travel-cost advantage than traveling by taxi in each functional zone both on weekdays and on weekends. Moreover, the top three functional zones with the biggest differences in the cost advantage of traveling by shared cars between weekdays and weekends are the functional zones of culture, financial and traffic.

Exploring the competitive advantages of car sharing in the space-time dimension and then understanding its influencing mechanism are of certain instructive significance for the planning and adjustment of both relevant government policies and enterprises’ operational strategies. To further improve the service ability and the utilization rate of car sharing resources and let car sharing play a better role in urban transportation, relevant departments should deeply understand the formation mechanism of the market demand for car sharing according to the focus markets on the travel-cost advantages of car sharing. On one hand, the government should provide more parking spaces reserved for car sharing only and should also increase subsidies for car sharing enterprises, which can alleviate parking problems and reduce parking charges. On the other hand, car sharing enterprises should set up more car sharing outlets, improve the convenience of car sharing, and optimize the user experience to make people accept car sharing well and to encourage more people to use shared cars.

In spite of the promise of the novel approach of comparing the travel-costs between car sharing and taxis, we must note a number of shortcomings that should be addressed in future work. Firstly, the car sharing service in Beijing is operated by several different enterprises such as the MoreFun platform owned by Beijing Automotive Group and the GoFun platform owned by Beijing Shouqi Group. But the car sharing orders used in this paper only comes from the MoreFun platform. Consequently, the results in our research might suffer sample bias issue when we analyze the quantitative distribution of car sharing stations and orders. The sharable cars that are operated by MoreFun platform are only used by a small number of travelers. Thus, the car sharing order data used in this paper could not fully show the travel characteristics of all car sharing users. Secondly, people’s travel behavior would be influenced by many factors such as travelers’ preference, weather conditions and the distribution of functional areas. However, there is a lack of information in our research data that describes individual characteristics such as people’s age, gender, occupation and income. Therefore, the results of this paper may not reflect the travel behavior characteristics of different user groups. Finally, we calculate the time cost according to traveler’s average income, which varies between people with different occupations. Additionally, in the process of calculating the fare of each car sharing order, we do not consider some special cases such as coupons or severe weather. The neglect of these special cases might result in a deviation of theoretical conclusions from actual situations.

In view of the above limitations, future work may focus on using a completer and more abundant dataset to generate more solid conclusions. Second, we may distinguish the travel behavior characteristics of different user groups based on the individual attributes such as age, occupation and gender. We will also consider travelers’ behavior during their travels such as whether to use coupons or charge shared cars. Thirdly, advanced statistical techniques are encouraged to be used to further investigate the dataset. Finally, this paper only compares the station-based car sharing with taxis. Future work would also focus on comparing car sharing with private car, bus and other transportation modes.

## Figures and Tables

**Table 1:** The comparison between car sharing, taxi, bus and subway.

	Car Sharing	Taxi	Bus	Subway
Service time	24-h service	24-h service	It is required to operate on fixed timetables and out of service at night	It is required to operate on fixed timetables and out of service at night
Price	High	High	Low	Low
Speed	Fast	Fast	Slow	Fast
Comfort	Travelers could sit the whole time	Passengers could sit the whole time	There are no empty seats sometimes and passengers have to stand often; It is crowded inside the vehicle sometimes	There are no empty seats sometimes and passengers have to stand often; It is crowded inside the vehicle sometimes
Flexibility	The routes are not fixed	The routes are not fixed	It is required to operate on fixed routes	It is required to operate on fixed routes
Privacy	Individuals enjoy high space privacy	Individuals enjoy relatively high space privacy	Individuals enjoy low space privacy	Individuals enjoy low space privacy
Accessibility	Travelers need to return the car to a fixed car sharing station which may be a distance from their destinations	Passengers can be taken to their destinations directly	Passengers need to get off at a fixed stop which may be a distance from their destinations	Passengers need to get off at a fixed stop which may be a distance from their destinations

**Table 2:** The research data.

**The Data Fields of a Car Sharing Order**		
**Data Field**	**Example**	
Order ID	17070210112314087756	
Pick-up time stamp	2017/09/26 22:40:00	
Drop-off time stamp	2017/09/27 01:20:00	
Pick-up station	The ground parking lot of Taishan Hotel	
Drop-off station	The ground parking lot of Taishan Hotel	
**The Data Fields of a Tracking Record**		
**Data Field**	**Example**	
Order ID	17070210112314087756	
Time stamp	2017/09/26 22:40:05	
Longitude	116.400632°E	
Latitude	39.990488°N	
Instantaneous speed	12.2 km/h	
**The Information About Car Sharing Stations**		
**Name**	**Longitude**	**Latitude**
The ground parking lot of Taishan Hotel	116.335795°E	40.055445°N
The parking lot of Wenjin International Apartment	116.333669°E	39.998997°N
The ground parking lot of Haide Hotel	116.523158°E	39.942206°N

**Notes:** We list three car sharing stations in Table 2 as examples. ID—identifier.

**Table 3:** Charge standard of car sharing in Beijing.

Category	Fare
Travel fare per unit of time	0.17 yuan/min (i.e., about 0.03 dollars/min)
Travel fare per unit of distance	1 yuan/km (i.e., about 0.15 dollars/km)

**Table 4:** Charge standard of taxi in Beijing.

Category	Fare
Base rate (0–3 km)	13 yuan (i.e., about 1.92 dollars)
Mileage fee	2.3 yuan/km (i.e., about 0.34 dollars/km)
Low speed fee	If the speed is less than 12 km/h: add additional 2 km’s rental per 5 min during the morning and evening rush hours; add 1 km’s rental during other time periods (exclude empty cruise fee)
Empty cruise fee	For the part which is over 15 km of one way that carrying passengers, add 50% of the basic unit price; for the round-trip of which the distance between starting point and finishing point is within 2 km (include 2 km), there is no empty cruise fee
Night-time charge	For 23:00 (include 23:00) to 5:00 next day (exclude 5:00) operation: add additional 20% of the basic unit price

Notes: The pricing standard of taxi in the table is issued by Beijing Municipal Commission of Development and Reform [49].

**Table 5:** Frequency distribution table of car sharing orders’ stay points.

Parking Duration of Stay Point	Frequency	Ratio
0–5 min	1,602,174	91.84%
5–10 min	33,386	1.91%
10–15 min	15,020	0.86%
15–20 min	11,051	0.63%
20–25 min	7,928	0.46%
25–30 min	5,926	0.34%
Over 30 min	69,086	3.96%

## Appendix A

The result of the single sample T-test that is performed on the travel-costs of car sharing and taxis when the travel distance is more than 50 km is shown in Table A1. The results in Table A1 show a *p*-value < 0.05, indicating that the statistical result is significant.

**Table A1:** The result of the single sample T-test.

	N	The Maximum Value	The Minimum Value	The Average Value	The Standard Deviation	T-Value	*p*-Value
**F_car sharing_**	17696	1184.264	62.074	249.049	183.580	180.466	0.000
**F_taxi_**	17696	3087.038	13.000	309.661	169.429	243.129	0.000

Notes: N is the number of car sharing orders whose travel distance is more than 50 km. *F_car sharing_* and *F_taxi_* denote the travel-cost of car sharing and that of taxis for each car sharing order, respectively.

The result of the paired-samples T-test that is performed on the difference of travel-costs between car sharing and taxis when the travel distance is more than 50 km is shown in Table A2. The results in Table A2 show a *p*-value < 0.05, indicating that there is a significant difference in travel-costs between car sharing and taxis. Besides, the average value in Table A2 indicates that travel-cost of taxis is far higher than that of car sharing when the travel distance is more than 50 km.

**Table A2:** The result of the paired-samples T-test.

	N	The Maximum Value	The Minimum Value	The Average Value	The Standard Deviation	T-Value	*p*-Value
**D_travel-cost_**	17696	2310.137	−966.148	60.657	162.675	49.601	0.000

Notes:*D_travel-cost_ = F_taxi_ - F_car sharing_*. *D_travel-cost_* denotes the difference of travel-costs between taxis and car sharing for each car sharing order.

The result of the single sample T-test that is performed on the travel-costs of car sharing and taxis when the travel time is from 20 to 60 min is shown in Table A3. The results in Table A3 show a *p*-value < 0.05, indicating that the statistical result is significant.

**Table A3:** The result of the single sample T-test.

	N	The Maximum Value	The Minimum Value	The Average Value	The Standard Deviation	T-Value	*p*-Value
**F_car sharing_**	3367	57.857	4.750	17.452	8.153	124.210	0.000
**F_taxi_**	3367	159.882	13.000	33.608	21.087	92.481	0.000

Notes: N is the number of car sharing orders whose travel time is from 20 to 60 min.

The result of the paired-samples T-test that is performed on the difference of travel-costs between car sharing and taxis when the travel time is from 20 to 60 min is shown in Table A4. The results in Table A4 show a *p*-value < 0.05, indicating that there is a significant difference in travel-costs between car sharing and taxis. Besides, the average value in Table A4 indicates that travel-cost of taxis is higher than that of car sharing when the travel time is from 20 to 60 min.

**Table A4:** The result of the paired-samples T-test.

	N	The Maximum Value	The Minimum Value	The Average value	The standard deviation	T-Value	*p*-Value
**D_travel-cost_**	3367	138.578	−44.314	16.156	15.626	59.994	0.000
